# Improvement of the thermostability and catalytic efficiency of a highly active β-glucanase from *Talaromyces leycettanus* JCM12802 by optimizing residual charge–charge interactions

**DOI:** 10.1186/s13068-016-0544-8

**Published:** 2016-06-13

**Authors:** Shuai You, Tao Tu, Lujia Zhang, Yuan Wang, Huoqing Huang, Rui Ma, Pengjun Shi, Yingguo Bai, Xiaoyun Su, Zhemin Lin, Huiying Luo, Bin Yao

**Affiliations:** Key Laboratory for Feed Biotechnology of the Ministry of Agriculture, Feed Research Institute, Chinese Academy of Agricultural Sciences, No. 12 Zhongguancun South Street, Beijing, 100081 People’s Republic of China; State Key Laboratory of Bioreactor Engineering, East China University of Science and Technology, Shanghai, 200237 People’s Republic of China; Institute of Animal Science and Veterinary Medicine, Hainan Academy of Agricultural Sciences, Haikou, 571100 People’s Republic of China

**Keywords:** *Talaromyces leycettanus* JCM12802, Endo-β-1,3-1,4-glucanase, Thermostability improvement, High specific activity, Charge–charge interaction

## Abstract

**Background:**

β-Glucanase is one of the most extensively used biocatalysts in biofuel, food and animal feed industries. However, the poor thermostability and low catalytic efficiency of most reported β-glucanases limit their applications. Currently, two strategies are used to overcome these bottlenecks, i.e., mining for novel enzymes from extremophiles and engineering existing enzymes.

**Results:**

A novel endo-β-1,3-1,4-glucanase of GH16 (*Tlglu16A*) from the thermophilic fungus *Talaromyces leycettanus* JCM12802 was produced in *Pichia pastoris* and characterized. For potential industrial applications, recombinant *Tl*Glu16A exhibits favorable enzymatic properties over most reported glucanases, i.e., remarkable stability over a wide pH range from 1.0 to 10.0 and superior activity on glucan substrates (up to 15,197 U/mg). The only weakness of *Tl*Glu16A is the thermolability at 65 °C and higher. To improve the thermostability, the enzyme thermal stability system was then used to engineer *Tl*Glu16A through optimization of residual charge–charge interactions. Eleven mutants were constructed and compared to the wild-type *Tl*Glu16A. Four mutants, H58D, E134R, D235G and D296K, showed longer half-life time at 80 °C (31, 7, 25, 22 vs. 0.5 min), and two mutants, D235G and D296K, had greater specific activities (158.2 and 122.2 %, respectively) and catalytic efficiencies (*k*_cat_/*K*_m_, 170 and 114 %, respectively).

**Conclusions:**

The engineered *Tl*Glu16A has great application potentials from the perspectives of enzyme yield and properties. Its thermostability and activity were apparently improved in the engineered enzymes through charge optimization. This study spans the genetic, functional and structural fields, and provides a combination of gene mining and protein engineering approaches for the systematic improvement of enzyme performance.

**Electronic supplementary material:**

The online version of this article (doi:10.1186/s13068-016-0544-8) contains supplementary material, which is available to authorized users.

## Background

β-Glucans are linear polymers of β-D-glycosyl residues and represent the most important constituent of endosperm cell walls in cereals (barley, wheat and rye) [1, 2]. Endoglucanases are the enzymes that specifically cleave the mixed (1 → 3) or (1 → 4) linkages of β-D-glucan [3] and are classified into endo-1,3-1,4-β-glucanase (lichenase; EC 3.2.1.73), endo-1,3-β-glucanase (laminarinase; EC 3.2.1.39) and endo-1,3(4)-β-glucanase (EC 3.2.1.6) based on their modes of action [4, 5]. β-Glucanases are important commercial biocatalysts in various industries. For example, the accretion of exogenous β-glucanases can reduce the undesirable effects of barley β-glucan in the process of mashing in the brewing industry and can enhance the β-glucan digestibility in poultry feedstuffs [6–8]; endoglucanase is also used in biomass conversion to bioethanol in combination with xylanase and has application potential in bioenergy production [9].

A large proportion of β-glucanases are unstable during the high-temperature processes. To improve the thermostability of glucanases, either mining new genetic resources of thermophiles, engineering the protein, or optimizing application procedures is the most common practice [10, 11]. Thermophilic bacteria like *Fibrobacter* [12], *Streptomyces* [13], *Bacillus* [14, 15] and *Alicyclobacillus* [16] have been reported to produce high-temperature active glucanases, and thermophilic fungi are another source of glucanase genes, such as *Paecilomyces* spp. [17], *Thermoascus aurantiacus* CBMAI-756 [18] and *Talaromyces leycettanus* [19]. The thermophilic *Talaromyces* spp. are known to be potential industrial enzyme producers, as they have been reported to secrete various kinds of hydrolytic enzymes such as mannanase [19] and α-galactosidase [20]. However, no β-glucanase of GH16 has been reported from this genus yet.

Protein engineering is an important tactic to acquire thermostable enzymes, including but not limited to augmenting the number of disulfide bridges, hydrogen bonds, or salt bridges, introducing ionic bonds [21] or cation–π interactions [22] and replacing the N terminus [23]. For example, Wang et al. [24] employed directed evolution to construct a hyperthermostable xylanase mutant with an increased half-life of >9 times (about 228 min); Wintrode et al. [25] utilized DNA shuffling to create a protease mutant with increased melting temperature (*T*_m_) of 25 °C and half-life improvement of 1200-fold at 60 °C. However, along with the improvement of enzyme thermostability, enzyme activity often decreases at various degrees. How to improve the enzyme properties without activity loss is always the biggest challenge.

Optimization of residual charge–charge interactions is a structure-based rational design approach that has been proven to be an effective method for thermostability improvement [26, 27]. However, its extensive application has been limited by underdeveloped bioinformatic tools. Tanford and Kirkwood [28] initially set up the TK model to calculate the contribution of each single charged residue to overall stability. This model was then improved by introducing solvent accessibility (SA) [29], Gibbs free energy [30] and electrostatic interactions [31]. Using a suite of enzyme redesign algorithms, enzyme thermal stability system (ETSS) [32] was developed to refine the calculation of the TK-SA model and surface charge–charge interaction analysis. By replacing positively charged residues with negatively charged or neutral ones, the *E*_*ij*_ value is decreased and the enzyme thermostability would be improved. A previous study has shown that ETSS is efficient in the improvement of thermostability and catalytic efficiency of a GH28 endopolygalacturonase [33]. In this study, we identified a novel endoglucanase of GH16 and employed the ETSS to engineer the protein for better industrial performance.

## Results and discussion

### Gene cloning and sequence analysis

A gene fragment, 553 bp in length, was amplified from the genomic DNA of *T. leycettanus* JCM12802 using the degenerate primers [34]. BLASTx analysis indicated that the 553 bp fragment had the uppermost deduced amino acid sequence identity (80 %) to an uncharacterized endo-β-glucanase of GH16 (XM013476233). The DNA and cDNA of the complete gene (*Tlglu16A*) were obtained by thermal asymmetric interlaced (TAIL)-PCR and RT-PCR, respectively. Sequence analysis manifested that the open reading frame (ORF) of endoglucanase gene (*Tlglu16A*) consists of 1198 bp that is interrupted by two introns (61 and 69 bp, respectively, in length). The deduced *Tl*Glu16A consists of 355 amino acid residues and shares the maximum identity of 76 % with a putative endo-1,3(4)-β-glucanase from *Rasamsonia emersonii* CBS 393.64 (KKA25075), 72 % identity with the functionally characterized endo-1,3(4)-β-glucanase from *Penicillium* sp. C1 (AFC38442) and 64 % identity with the structurally resolved glucanase from *Paecilomyces thermophila* (3WDT). SignalP analysis indicated that the deduced *Tl*Glu16A had a predicted 20-residue signal peptide. The *p*I value and the calculated molecular mass of the mature protein were estimated to be 4.69 and 35.5 kDa, respectively. Two predicted N-glycosylation sites, Asn277 and Asn309, were identified. The deduced *Tl*Glu16A harbors a single module, the catalytic domain of GH16. The conserved motif EIDIIE of GH16 fungal glucanases was identified, and Glu112 and Glu117 were the conjectural nucleophile and acid/base catalytic residues, respectively (Additional file 1).

### Expression, purification and identification of *Tl*Glu16A

*Escherichia coli*, *Bacillus subtilis*, *Pichia pastoris*, *Saccharomyces cerevisiae*, *Aspergillus oryzae* and *Trichoderma reesei* represent the most widely used heterologous expression systems [35]. Of them, the *P. pastoris* system is strong as a powerful promoter, in the secretion of eukaryotic proteins, correct folding of heterologous proteins and high-level expression at low cost [36, 37]. Using this system, a large number of glucanases have been successfully expressed in *P. pastoris* [38]. In this study, we produced recombinant *Tl*Glu16A in *P. pastoris* GS115 competent cells. After 48 h induction with methanol at 30 °C, a transformant showing the highest β-glucanase activity on barley β-glucan (tested at pH 5.0, 50 °C and 10 min) in 1.5 mL tube culture was selected for high cell density fermentation in a 15-L fermentor. The β-glucanase activity reached 22,450 U/mL after 144 h of methanol induction. The total secreted proteins reached a relatively high titer of 1.64 g/L, 90 %, which was verified to be recombinant *Tl*Glu16A (data not shown). This expression level was greater than that of β-glucanases produced in *P. pastoris*, including Bgl7A (370 mg/L) from *Bispora* sp. MEY-1 [39], Bgl (0.8 g/L) from *Paenibacillus* sp. F-40 [40] and even the codon optimized β-1,3-1,4-glucanase (250 mg/L) from *B. licheniformis* [15].

Recombinant *Tl*Glu16A in the culture supernatants was purified to electrophoretic homogeneity by anion exchange chromatography. The purified enzyme showed a single band with an apparent molecular mass of 43 kDa on SDS-PAGE (Additional file 2), which is higher than the calculated molecular weight of the protein (35. 5 kDa). Three peptides of the purified recombinant *Tl*Glu16A, DHTNVASGSGR, GSIPADISSGS and VYQDTAEST, were identified by the MALDI-TOF/MS analysis, which completely corresponded to the deduced amino acid sequence of *Tl*Glu16A. The result indicates that the single band on SDS-PAGE is purified recombinant *Tl*Glu16A indeed, and N-glycosylation might occur in *Tl*Glu16A during heterologous expression in *P. pastoris*. After deglycosylation with Endo H, recombinant *Tl*Glu16A showed a molecular mass of 35 kDa, which is consistent with the predicted size.

### Enzymatic properties of purified recombinant *Tl*Glu16A

**Fig. 1:**
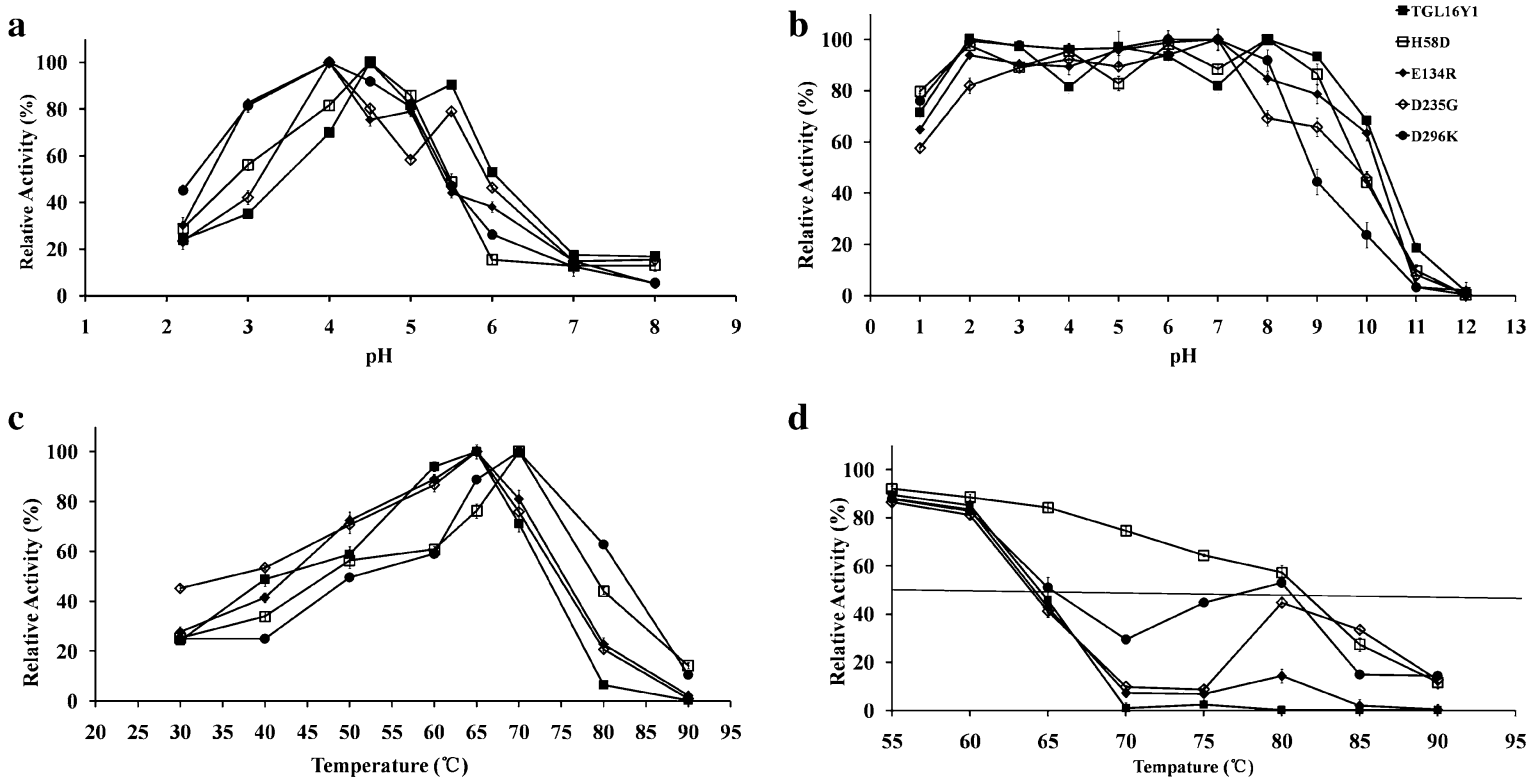
Enzymatic properties of wild-type *Tl*Glu16A and its mutants. **a** pH-dependent activity profiles. **b** pH stability. **c** Temperature-dependent activity profiles. **d** Enzyme inactivation at different temperatures for 30 min

All β-glucanase activities were assayed with barley β-glucan as the substrate. The purified *Tl*Glu16A displayed the optimal activity at pH 4.5 when assayed at 60 °C for 10 min (Fig. 1a), which falls within the pH range of fungal β-glucanases (pH 4.0–7.0) from *Cochliobolus carbonum* [41], *T. emersonii* [42], *Rhizopus microsporus* var. *microsporus* [6] and *P. thermophile* [43]. Nevertheless, the pH stability of *Tl*Glu16A is broader than most bacterial β-1,3-1,4-glucanases (pH 6.0–7.5). It retained more than 65 % of its original activity after being incubated in different buffers with a pH range of 1.0–10 (Fig. 1b) at 37 °C for 1 h. Therefore, *Tl*Glu16A would remain highly active and stable in the digestive tract of animals.

**Fig. 2:**
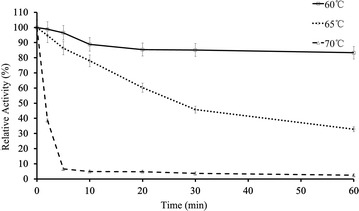
Thermostability of *Tl*Glu16A at 60, 65 and 70 °C

The optimal temperature of most reported β-1,3-1,4-glucanases ranges from 40 to 65 °C [4, 6, 8]. The optimal temperature of *Tl*Glu16A was determined to be 65 °C on barley β-glucan in 50 mM citrate buffer (pH 4.5) (Fig. 1c), only a little lower than that of β-glucanases from *Laetiporus sulphureus* var. *miniatus* [44] and *P. thermophile* [43]. The enzyme exhibited good thermostability at 60 °C for 1 h (Fig. 2), but lost activity rapidly with the half-lives of 25, 3, 1 and 0.5 min at 65, 70, 75 and 80 °C (Table 1), respectively. Most fungal β-1,3-1,4-glucanases are denatured at 65 °C and higher, such as the enzymes from *R. microsporus var. microsporus* (<1 min at 80 °C) [6], *P. thermophila* (13 min at 80 °C) [17] and *T. emersonii* CBS 814.70 (25 min at 80 °C) [45]. The only known exception is the β-1,3-1,4-glucanase from *L. sulphureus* var. *miniatus* that has a half-life of 60 min at 80 °C [7]. Thermostability is an essential parameter of enzymes for utilization in high-temperature industries (50–70 °C for the malting and 65–90 °C for feed pelleting) [7]. From the industrial point of view, the thermostability of *Tl*Glu16A should be improved.

**Table 1:** Half-lives of wild-type *Tl*Glu16A and its mutants for thermal inactivation

Enzyme	*t*_1/2_ (min) at			
	65 °C	70 °C	75 °C	80 °C
*Tl*Glu16A	25	3	1	0.5
H58D	77	56	37	31
E134R	41	3	4.5	7
D235G	39	4	3.5	25
D296K	60	15	30	22

The enzyme activity was assayed at each optimal condition for 10 min

The influences of chemical reagents and metal ions on the *Tl*Glu16A activity were tested at concentrations of 5 and 10 mM. Except for Ag^+^, Mn^2+^, Cu^2+^ and SDS that partially inhibited the glucanase activity of *Tl*Glu16A (Table 2), most chemicals had no effect on the *Tl*Glu16A activity. Of them, β-mercaptoethanol enhanced the enzymatic activity up to 1.30- and 1.46-fold at both tested concentrations. Co^2+^ and EDTA reinforced the β-glucanase activity at the concentration of 10 mM by 1.21- and 1.20-fold, respectively, which was different from most β-glucanases. The great resistance to most metal ions and chemicals widens the potential application spectrum of *Tl*Glu16A in more fields such as detergent and paper industries.

**Table 2:** Effects of metal ions and chemical reagents on the *Tl*Glu16A activity

Chemical	Relative activity (%)^a^		Chemical	Relative activity (%)	
	5 mM	10 mM		5 mM	10 mM
None	100.0 ± 0.9	100.0 ± 0.8	Fe^3+^	106.2 ± 2.1	99.6 ± 1.9
Na^+^	110.8 ± 1.8	120.4 ± 1.5	Pb^2+^	95.6 ± 2.7	91.8 ± 2.8
Co^2+^	116.6 ± 1.8	121.2 ± 1.7	Mn^2+^	87.2 ± 1.2	66.1 ± 2.1
K^+^	109.3 ± 2.1	119.6 ± 2.7	Cu^2+^	86.1 ± 2.4	48.5 ± 1.8
Ca^2+^	108.8 ± 1.3	114.9 ± 1.3	Ag^+^	78.4 ± 1.9	1.2 ± 0.7
Ni^2+^	108.3 ± 1.6	117.7 ± 2.1	β-Mercaptoethanol	130.8 ± 3.0	146.3 ± 2.4
Mg^2+^	106.5 ± 1.7	112.0 ± 2.1	EDTA	110.3 ± 2.5	120.0 ± 2.1
Cr^3+^	103.1 ± 1.6	116.0 ± 1.2	SDS	64.7 ± 3.1	64.6 ± 1.0
Zn^2+^	101.4 ± 2.1	111.3 ± 1.8			

^a^Values represent mean ± SD (*n* = 3) relative to the untreated control samples

*Tl*Glu16A had high specific activities toward barley β-glucan (15,197 ± 153 U/mg), lichenan (12,770 ± 98 U/mg) and laminarin (763 ± 87 U/mg), higher than the counterparts from *P. thermophila* (11,938 U/mg) [17], *P. pinophilum* C1 (12,622 U/mg) [34], *Paecilomyces* sp. FLH30 (8649 U/mg) [46], *F. succinogenes* (5180 U/mg) [37], *Bispora* sp. MEY-1 (4040 U/mg) [39], *Paenibacillus* sp. F-40 (3076 U/mg) [40], *B. subtilis* MA139 (728.79 U/mg) [21], and *Aspergillus niger* (63.83 U/mg) [22] with barley β-glucan as the substrate. Considering its great specific activity and high expression level as well as simple processing procedure, *Tl*Glu16A could be a desirable candidate for use in industrial applications with broad applicability.

### Substrate specificity, hydrolysis properties and kinetics of *Tl*Glu16A

The specific activities of the purified recombinant *Tl*Glu16A toward different substrates were determined. Similar to most fungal β-1,3-1,4-glucanases from thermophilic *R. miehei* [47], *A. japonicas* [48], *Melanocarpus* sp. [49] and *R. microsporus* var. *microsporus* [6], *Tl*Glu16A exhibited rigid substrate specificity (lichenan, barley β-glucan and laminarin), and had no activity on carboxymethyl cellulose-sodium (CMC-Na), carob bean gum, Avicel, 4-nitrophenyl β-d-cellobioside (*p*NPC), 4-nitrophenyl-α-d-galactopyranoside (*p*NPG) and birchwood xylan. Thus, the enzyme was classified as a β-1,3-1,4-glucanase on the strength of its substrate specificity. However, *Tl*Glu16A differs from the β-1,3-1,4-glucanases from *Laetiporus sulphureus* var. *miniatus* and *R. miehei* DSM 1330 that exhibited a higher relative activity toward laminarin (15–33 vs. 5 % against that of barley β-glucan) [50].

The kinetic values of *Tl*Glu16A for barley β-glucan and lichenan are shown in Table 3. The substrate affinity of *Tl*Glu16A (5.74 mg/mL) is similar to that of the β-glucanases produced by thermoacidophilic *Alicyclobacillus* sp. A4 [16] and *B. licheniformis* UEB CF [50], much lower than that of the β-1,3-1,4-glucanase from *R. microsporus* var. *microsporus* (19.8 mg/mL) [6], but higher than those of the β-1,3-1,4-glucanases from thermophilic *Malbranchea cinnamomea* [43] and *R. miehei* [42].

**Table 3:** Specific activities and kinetics of wild-type *Tl*Glu16A and its mutants with barley β-glucan and lichenan as the substrate

Enzymes	Barley β-glucan				Lichenan			
	Specific activity (U/mg)	*V*_max_ (μmol/min·mg^)^	*V*_max_ (μmol/min·mg^)^	*k*_cat_/*K*_m_ (mL/s·mg)	Specific activity (U/mg)	*K*_m_ (mg/mL)	*K*_m_ (mg/mL)	*K*_cat_/*K*_m_ (mL/s·mg)
*Tl*Glu16A	15,197 ± 153	5.74 ± 0.63	38,314 ± 187	3944 ± 156	12,770 ± 98	3.69 ± 0.54	13,447 ± 112	3643 ± 115
H58D	15,066 ± 113	2.52 ± 0.75	22,936 ± 113	5384 ± 127	13,408 ± 87	3.45 ± 0.46	27,100 ± 198	4637 ± 99
E134R	11,996 ± 79	5.04 ± 0.76	26,810 ± 154	3144 ± 143	7715 ± 88	2.64 ± 0.93	13,351 ± 167	2988 ± 198
D235G	24,040 ± 102	5.41 ± 1.01	61,350 ± 187	6701 ± 154	13,886 ± 102	9.32 ± 0.98	50,761 ± 201	3221 ± 154
D296K	18,574 ± 103	3.35 ± 0.43	25,381 ± 201	4479 ± 163	14,488 ± 101	7.39 ± 1.41	22,669 ± 175	3067 ± 103

Values are means ± standard deviations

**Fig. 3:**
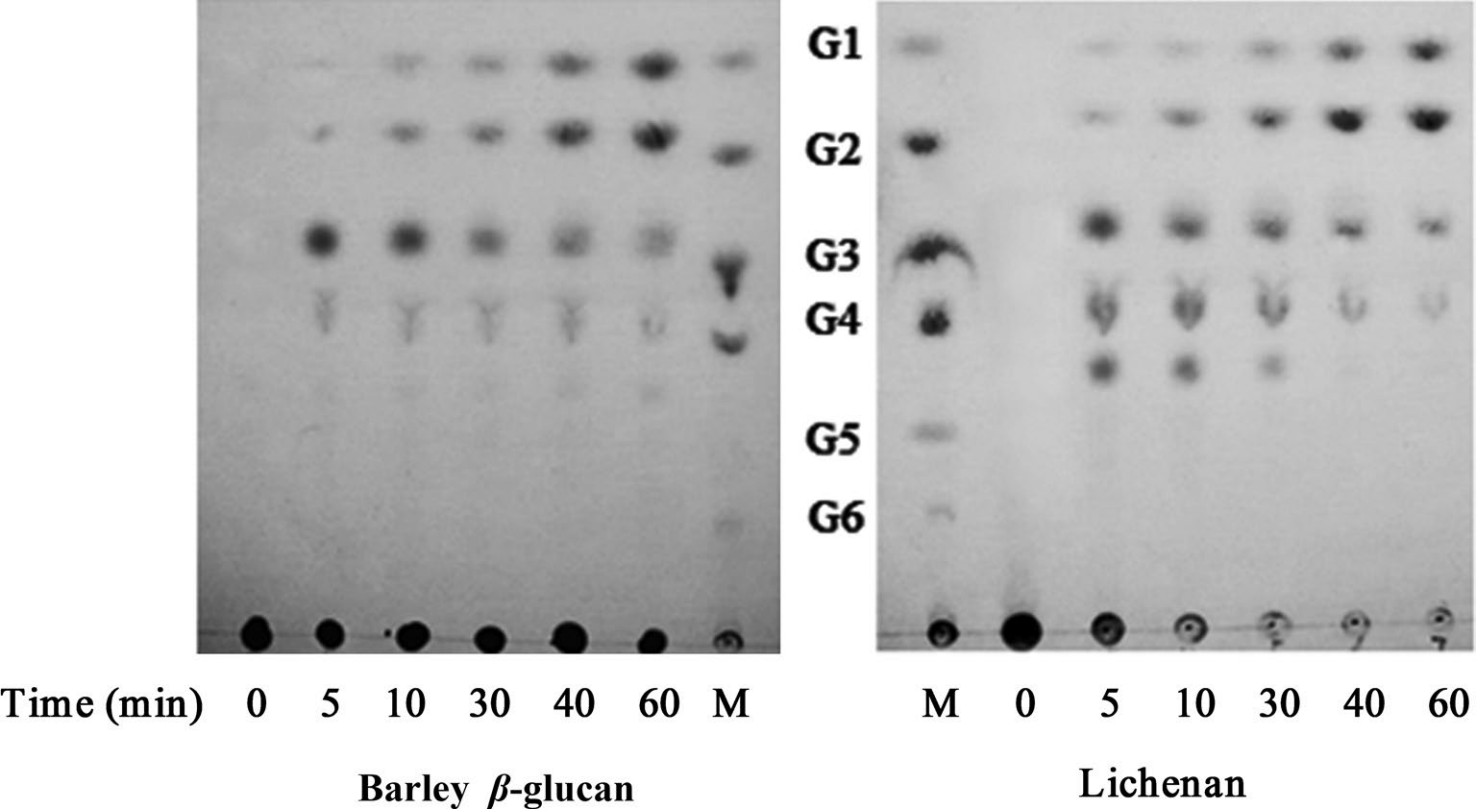
TLC analysis of the hydrolytic products of barley β-glucan and lichenan by purified *Tl*Glu16A. Glucose (*G1*), cellobiose (*G2*), cellotriose (*G3*), cellotetraose (*G4*), cellopentaose (*G5*) and cellohexaose (*G6*) were used as the standards (*M*)

The hydrolytic degradation products of barley β-glucan and lichenan by *Tl*Glu16A were exhibited on thin layer chromatography (TLC) (Fig. 3). Tetrasaccharide and trisaccharide were the main products at the initial stage (10 min). Along with the extended reaction time, trisaccharide and tetrasaccharide were further hydrolyzed into monosaccharides and disaccharides. In combination with the hydrolysis products, it suggested that *Tl*Glu16A had an endotype mode of action, and the enzyme is expected to be an endo-β-1,3-1,4-glucanase that specifically cleaves the β-1,4 linkages adjacent to β-1,3 glycosidic bonds and generates primarily tetrasaccharide, trisaccharide and disaccharide [42, 43].

In view of its optimal activity at acidic and high expression level, mesothermal conditions and high specific activity as well as simple processing method, *Tl*Glu16A would represent an excellent candidate for utilization in the animal feed industry.

### Selection of the mutagenesis sites in *Tl*Glu16A

**Fig. 4:**
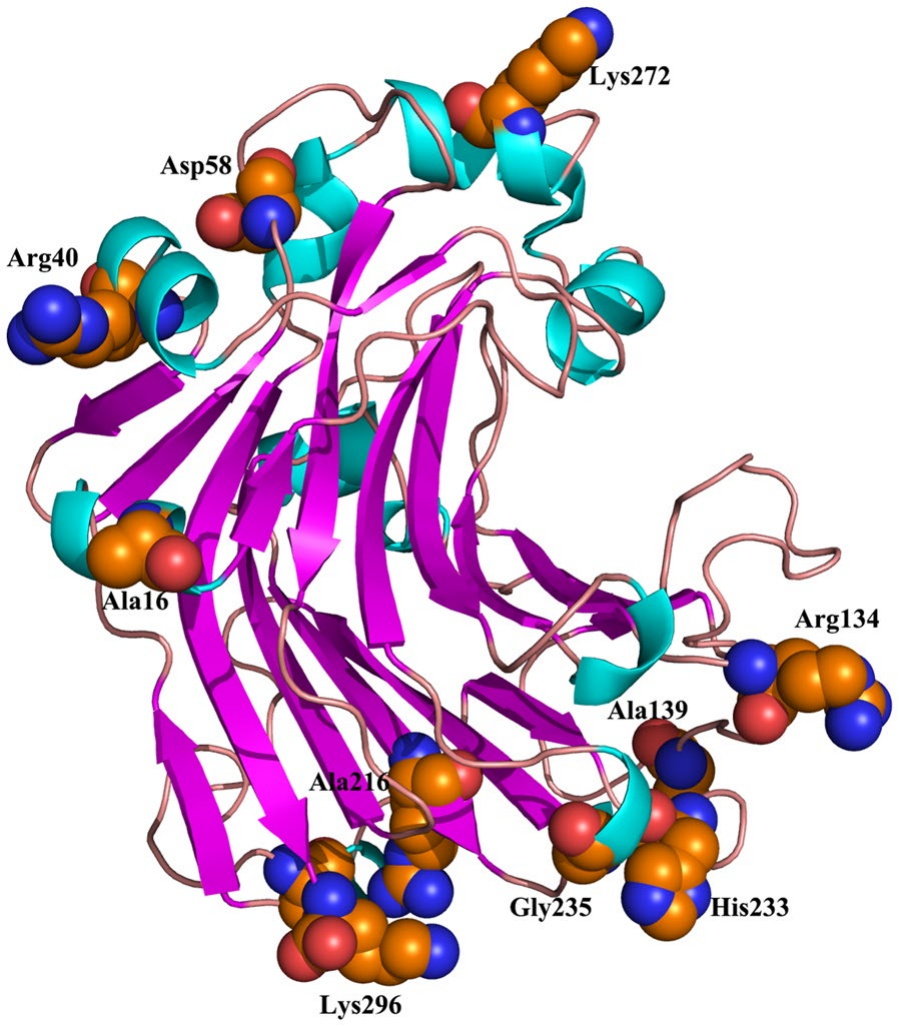
The modeled structure of *Tl*Glu16A viewed from the N-terminal side. The mutated sites are indicated with balls

ETSS calculation of total interaction energy (*E*_*ij*_) between charged amino acids of point *i* and *j* of wild-type *Tl*Glu16A was conducted (Additional file 3). Mutation sites were selected following the three criteria [27] as shown below: (1) residues with high positive *E*_*ij*_ values were taken as priorities; (2) prior residues far from the catalytic center were preferred to retain the activity; and (3) multiple-sequence alignment was combined to determine the mutation sites. As results, 23 residues with high positive *E*_*ij*_ values were identified, and 11 far from the catalytic center were selected for site-directed mutagenesis based on the multiple-sequence alignment. Among them, three residues were mutated to neutral A (D16A, D139A and D216A), five residues to positively charged R or K (E40R, E134R, E190R, D272K and D296K), and three to D, H or G (H58D, D233H, and D235G). All of them were far away from the catalytic center (partial data shown in Fig. 4). On account of the ETSS analysis, when H58, E134 and D296 were mutated to D, R and K, respectively, the *E*_*ij*_ values decreased from 2.3 to 1.0, 8.0 to −7.7 and 2.3 to −3.1 kJ/mol, respectively. These modifications converted the enzyme into a more stable state and improved the thermostability accordingly.

### Generation, expression and purification of wild-type *Tl*Glu16A and its mutants

The 11 site-directed mutants of *Tl*Glu16A were constructed and expressed in *P. pastoris*, as described herein. Among them, mutants H58D, E134R, D235G and D296K showed greatly increased thermostability and maintained substantial activity (data not shown). These four mutants were then purified. SDS-PAGE analysis revealed that the four mutants had molecular masses of 43–55 kDa. After treatment with Endo H, all enzymes appeared as a single band in agreement with the theoretical mass of 35.5 kDa (Additional file 2).

### Comparison of the properties of *Tl*Glu16A and its mutants

Wild-type *Tl*Glu16A and its mutants showed little difference in the pH activity profiles at the pH range of 5.5–7.0 (Fig. 1a). Compared with that of *Tl*Glu16A, the pH adaptability ranges of the mutants shifted to acidic, and the activities of mutants H58D, E134R, D235G and D296K at pH 3.0 showed an increase of 21, 47, 7 and 46 %, respectively. The mutant enzymes were highly stable at pH 2.0–9.0 as *Tl*Glu16A (Fig. 1b). This may benefit the utilization of this protein in the animal feed industry. The four mutants displayed increased activity at higher temperatures (Fig. 1c). The temperature optima of mutants H58D and D296K were both at 70 °C, which was 5 °C higher than that of the original *Tl*Glu16A (65 °C). Although the temperature optima of E134R and D235G were the same as wild-type *Tl*Glu16A, their activity beyond 60 °C decreased slightly more than that of *Tl*Glu16A. At 70 °C, all mutants displayed above 76 % maximal activity, which was higher than that of wild-type *Tl*Glu16A (71 % of maximal activity). When the temperature was increased to 80 °C, all mutants showed above 20 % maximal activity, especially D296K and H58D maintained 62 and 44 % of the activity, respectively, which was much higher than that of wild-type *Tl*Glu16A (6.0 % of maximal activity). This indicates that H58D and D296K are more thermotolerant than the wild-type and other mutants.

*T*_50_ determination is a useful method to estimate and directly compare the thermostabilities of enzymes. As demonstrated in Fig. 1d, the *T*_50_ values of wild-type *Tl*Glu16A and its mutants were determined at the temperature range of 55–90 °C. The *T*_50_ value of wild-type *Tl*Glu16A was confirmed to be 64.5 °C, while that of mutant enzymes H58D and D296K was increased by 16.8 and 0.8 °C. The *T*_50_ value of the other two mutants E134R and D235G were 64.2 and 64.0 °C. But the three mutants (E134R, D235G and D296K) had remarkable thermostability improvement at 80 °C (the temperature of feed pelleting) and *Tl*Glu16A almost completely lost the glucanase activity, while E134R, D235G and D296K retained 14, 44 and 52 % of the initial activity. It is strange that these three mutants retained more activity after incubation at 80 °C than at 75 and 70 °C. This phenomenon has been observed in lysozyme [53] and might be ascribed to the protein renaturation at higher temperature. The underlying mechanism might be that the changes of protein surface charge influence the three-dimensional structure of protein folding. GH16 endo-1,3-1,4-β-glucanase has a β-sandwich structure formed by two large anti-parallel β-sheets assembled face to face, which probably more easily renatures after incubation at high temperature, but the detailed mechanism is not clear.

Thermostabilities of recombinant *Tl*Glu16A and its mutants were estimated by their half-lives (*t*_1/2_) (Table 1). Compared with wild-type *Tl*Glu16A, all mutants have significant improvements in thermostability as shown in *T*_50_. The mutant E134R had a marginally increased *t*_1/2_ value (0.6- to 3.5-fold) at 65–75 °C. The D235G was much more thermostable, with an increased *t*_1/2_ value up to 49-fold at 80 °C. The mutants H58D manifested the maximal improvement in thermostability, with *t*_1/2_ increased up to 2-fold at 65 °C, 17-fold at 70 °C, 36-fold at 75 °C and 61-fold at 80 °C. This result also suggests that renaturation may occur in the mutants.

**Fig. 5:**
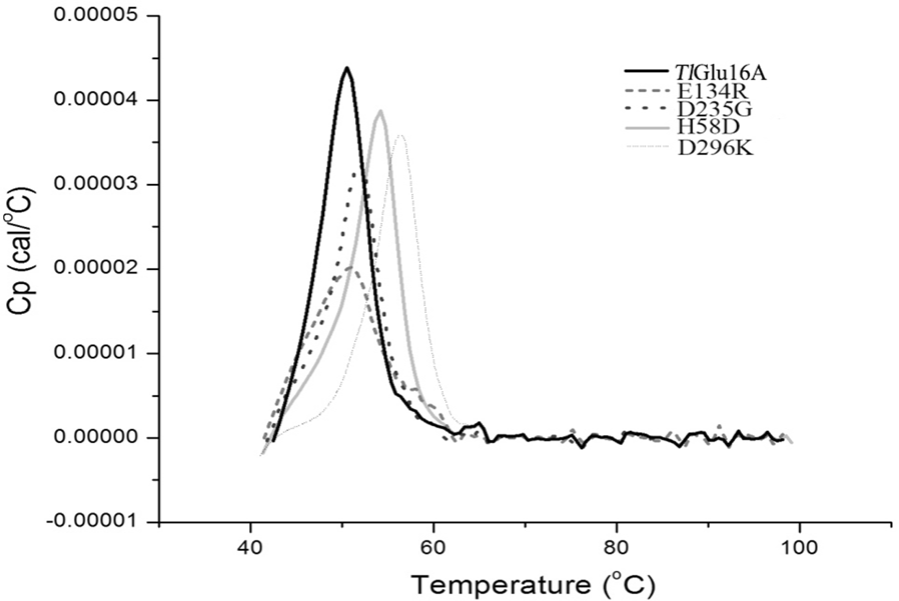
Thermograms of *Tl*Glu16A and its mutants detected using the DSC. The calorimetric recordings were scanned at 1 °C/min in 10 mM PBS (pH 6.8) with the protein concentration of 350 μg/mL, respectively

Differential scanning calorimetry (DSC) was executed to measure the *T*_m_ values of wild-type *Tl*Glu16A and its mutants over the temperature range of 40–100 °C (Fig. 5). Compared to the *T*_m_ of *Tl*Glu16A (50.55 °C), the *T*_m_ values of mutants E134R, D235G, H58D and D296K showed an increase of ~0.6 °C (51.14 °C), 1.5 °C (52.02 °C), 3.6 °C (54.19 °C) and 6.0 °C (56.49 °C), respectively. These data support that residue substitutions at sites 58 and 296 make the most outstanding contributions to thermostability improvement.

Under optimum conditions, the specific activities of the four mutants were comparable or higher than that of wild-type *Tl*Glu16A (15,197 U/mg) (Table 3). The catalytic activities and efficiency of the mutants D235G and D296K generated in this work were higher than the wild-type *Tl*Glu16A, and compared to the wild-type *Tl*Glu16A, the specific activities of mutant enzymes D235G and D296K were increased by 58.2 and 22.2 %, respectively, with barley β-glucan as the substrate. The *K*_m_ and *k*_cat_/*K*_m_ of *Tl*Glu16A are 5.74 mg/mL and 3944 mL/s mg, respectively. Mutant D235G increased the *k*_cat_ value almost twofold. In contrast, mutant D296K increased the affinity toward β-glucan. As a result, the *k*_cat_/*K*_m_ value of mutants D235G and D296K were onefold to twofold more compared to wild-type *Tl*Glu16A. Enzyme activity had been sacrificed for improved thermostability. In contrast, our main objective in this work is to improve the enzyme catalytic efficiency while improving thermal stability. Compared with the wild-type *Tl*Glu16A, the specific activities of mutant enzymes D235G and D296K were increased by 58.2 and 22.2 %, respectively, when using β-glucan as the substrate. This study reveals that the residue charge–charge interaction plays important roles in stabilizing enzymes; in addition, we managed to improve the enzyme thermostability accompanied by enhanced catalytic efficiency. It was a very rare find in the field of thermostable study. The great care in selection of mutation sites far away from the catalytic core was successful (Fig. 4).

Interest in feed enzymes is high, so thermophilic/thermostable glucanases have been mined from diverse species, cloned and characterized. However, few glucanases are economically viable for feed applications due to limitation in activity and stability under the compulsory processing conditions (70–80 °C for 5 min). Therefore, glucanases with higher stability and activity under the feed processing conditions are of great value. In this study, two mutants H58D and D296K have a remarkable thermostability at 80 °C, and two mutants D235G and D296K exhibit higher specific activity and better thermostability than *Tl*Glu16A.

**Fig. 6:**
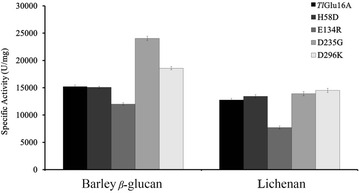
Activity profiles of *Tl*Glu16A and its mutants with barley β-glucan and lichenan as the substrates

When the catalytic efficiency of an enzyme increases, the protein structure becomes more flexible. Consequently, the rate of the substrate into the passage and product from the catalytic center will increase, and the thermostability of the protein may be reduced [51]. However, in this study, we not only increased the thermostability of *Tl*Glu16A, but also enhanced its catalytic efficiency and specific activity (Fig. 6) through modifying the charge–charge interaction of the protein surface. It is of importance to reveal that the surface charge of a protein plays key roles in protein thermostability and catalytic performance.

### Enzymatic hydrolysis of corn stover

**Fig. 7:**
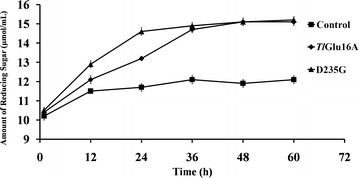
The hydrolysis of pretreated corn stover by wild-type *Tl*Glu16A and mutant D235G (each 0.06 μmol) at 50 °C and pH 4.8

Corn stover is composed of cellulose, hemicellulose, and lignin. When incubated at pH 4.8 and 50 °C with agitation, reducing sugars are released from the corn stover under study (Fig. 7). Without enzymatic addition, less than 12 μmol/mL of reducing sugars were released from the corn stover over 60 h. In contrast, the addition of wild-type *Tl*Glu16A and mutant enzyme H58D improved the hydrolysis efficiency, increasing the amounts of reducing sugar to 13.3 and 14.6 μmol/mL, respectively, at 24 h. When lengthening the incubation period to 36 h and longer, no difference was detected between *Tl*Glu16A and H58D. By hydrolyzing the β-1,4 bonds of hemicellulose, *Tl*Glu16A and its mutant H58D may assist in the efficient degradation of corn stover.

## Conclusions

In this study, we mined a thermophilic and highly active endo-1,3-1,4-β-glucanase *Tl*Glu16A from the thermophilic fungus *T. leycettanus* JCM12802. *Tl*Glu16A has a broad pH stability range, wonderful resistance to most metal ions and chemical reagents, and great enzymatic activity against synthesized and natural substrates. By optimizing the residual charge–charge interactions, the thermostability and catalytic efficiency of *Tl*Glu16A were remarkably and simultaneously improved. This study provides an excellent glucanase candidate in wide industrial applications and indicates the significance of charge–charge interactions in protein stabilization.

## Methods

### Strains, materials and culture medium

*Talaromyces leycettanus* JCM12802 was purchased from the Japan Collection of Microorganisms RIKEN BioResource Center (Tsukuba, Japan). *E. coli* Trans1-T1, vector pEASY-T3, the DNA purification kit, LA Taq DNA polymerase and Genome Walking kit were purchased from TaKaRa (Tsu, Japan). The RNA isolation system kit was purchased from Promega (Madison, WI, USA). Restriction endonucleases, T_4_ DNA ligase and Endo H (endo-β-*N*-acetylglucosaminidase H) were purchased from New England Biolabs (Ipswich, MA). The DNA isolation kit and *pfu* DNA polymerase were purchased from Tiangen (Beijing, China). The substrates barley β-glucan, lichenan, laminarin, CMC-Na, birchwood xylan, Avicel, *p*NPG, *p*NPC and carob bean gum were purchased from Sigma-Aldrich (St. Louis, MO). The plasmid pPIC9 and *P. pastoris* GS115 (Invitrogen, Carlsbad, CA) were used as the gene expression vector and expression host strain, respectively. The low-molecular-weight calibration kit was purchased from GE Healthcare (Pittsburgh, PA). Minimal dextrose medium (MD), buffered glycerol-complex medium (BMGY) and buffered methanol-complex medium (BMMY) were prepared as described in the instructions of the *Pichia* Expression Kit (Invitrogen). All chemicals were of analytical grade and commercially available.

### Gene cloning

The total genomic DNA of *T. leycettanus* JCM12802 was extracted using the Omega Fungal DNA Mini kit (Norcross, GA) after 72 h of shake cultivation in potato dextrose broth at 40 °C and used as template for PCR. A primer set specific for fungal GH16 β-glucanases (GH16F and GH16R) [34] was used to amplify the core region. The final PCR products (~550 bp) were purified, linked with pEASY-T_3_ vector and then transformed into *E. coli* Trans1-T1 for sequencing. TAIL-PCR [52] was conducted with the TaKaRa Genome Walking kit and six nested insertion-specific primers (Additional file 4) to obtain the 5′ and 3′ flanking regions of the core region. The flanking regions were sequenced and assembled with the core region sequence to give the full-length gene (*Tlglu16A*).

To induce β-glucanase production, *T. leycettanus* JCM12802 was cultured in a medium containing 5 g/L (NH_4_)_2_SO_4_, 1 g/L KH_2_PO_4_, 500 mg/L MgSO_4_·7H_2_O, 200 mg/L CaCl_2_, 10 mg/L FeSO_4_·7H_2_O and 30 g/L corn straw, pH 4.0 at 40 °C for 3 days. Total RNA was extracted using the Promega SV Total RNA Isolation System, and the cDNA was synthesized with a reverse transcription kit (TOYOBO, Osaka, Japan). The full-length cDNA of *Tlglu16A* was amplified using the specific primers Tlglu16A-F and Tlglu16A-R (Additional file 4). The PCR products were purified, linked with pEASY-T3 vector and then transformed into *E. coli* Trans-T1 for sequencing.

### Sequence analysis and structure modeling of *Tl*Glu16A

Nucleotide and amino acid sequences were analyzed using the BLASTx and BLASTp programs [53] (http://www.ncbi.nlm.nih.gov/BLAST/) in combination with the homology search. The ORF was identified using the program ORF Finder from the National Center for Biotechnology Information (NCBI). Vector NTI 10.0 software (Invitrogen) was used to forecast the molecular mass of the mature protein. Multiple-sequence alignment was analyzed with ClustalX and rendered by the ESPript3.0 program (http://espript.ibcp.fr/ESPript/cgi-bin/ESPript.cgi). The SignalP 4.0 server (http://www.cbs.dtu.dk/services/SignalP/) was used to predict signal peptide. The N- and O-glycosylation sites were predicted using online programs NetNGlyc 1.0 Server (http://www.cbs.dtu.dk/services/NetNGlyc/) and NetOGlyc 4.0 Server (http://www.cbs.dtu.dk/services/NetOGlyc/), respectively. Based on the known crystal structure of a GH16 β-glucanase from *P. thermophila* (3WDT), the 3-D structures of *Tl*Glu16A and its mutants were homologically modeled using the Swiss-model program (http://www.swissmodel.expasy.org/).

### Protein expression, purification and identification

The gene fragment encoding mature *Tl*Glu16A was amplified by PCR with the primers (Additional file 4) and then cloned into the vector pPIC9 at the *Eco*RI and *Not*I restriction sites. Transformed plasmids were electroporated into competent *P. pastoris* GS115 cells following the manufacturer’s instructions (Invitrogen). Ninety-six individual *Pichia* clones were selected and screened for β-glucanase activity on 0.5 % barley β-glucan as described by Chen et al. [34]. Those exhibiting the highest β-glucanase activity were subjected to high cell density fermentation in a 15-L fermenter (pH 5.0 and 30 °C) on the basis of the manufacturer’s instructions (Invitrogen). β-Glucanase production in culture supernatant was monitored every 24 h by glucanase activity assay until 144 h. The crude enzyme was loaded onto the HiTrap TM Desalting column and HiTrap TM Q Sepharose XL 5-mL FPLC column (GE Healthcare, Uppsala, Sweden) that were both equilibrated with 20 mM McIlvaine buffer (pH 6.8). Proteins were eluted using a linear gradient of NaCl (0–1.0 M) at a flow rate of 2.5 mL/min. Fractions were collected, assayed for enzyme activity and those having glucanase activities were subjected to sodium dodecyl sulfate-polyacrylamide gel electrophoresis (SDS-PAGE) as described by Laemmli [54]. Liquid chromatography/electrospray ionization tandem time-of-flight mass spectrometry (LC/ESI-TOF-MS) analysis of the trypsin-digested protein was conducted at the Institute of Zoology, Chinese Academy of Sciences.

The purified recombinant *Tl*Glu16A was deglycosylated by Endo H at 37 °C for 2 h according to the manufacturer’s specifications. The deglycosylated enzyme was also analyzed by SDS-PAGE.

### Glucanase activity assay

The glucanase activity was analyzed by measuring the release of reducing sugar from barley β-glucan and lichenan with 3,5-dinitrosalicylic acid (DNS) [55]. The standard reaction mixture system consisting of 100 μL of appropriately diluted enzyme and 900 μL of substrate solution (0.5 % [wt/vol] barley β-glucan or lichenan in McIlvaine buffer [200 mM Na_2_HPO_4_, 100 mM citric acid, pH4.0]) was incubated at 60 °C for 10 min. The reaction was terminated with 1.5 mL of DNS reagent. One unit of glucanase activity was defined as the amount of enzyme that produced 1 μmol of reducing sugar per minute. Each reaction and its controls were run in triplicate.

### Biochemical characterization of recombinant *Tl*Glu16A

The pH profile of *Tl*Glu16A was determined by measuring the glucanase activity after incubation at 60 °C in McIlvaine buffer (pH 2.2–8.0) containing 0.5 % barley β-glucan for 10 min. To determine the temperature for maximal activity, enzyme activity was measured at optimal pH and temperatures between 30 and 90 °C in McIlvaine buffer (pH 4.0) for 10 min. To verify pH stability, the enzyme was preincubated in buffers of various pH values (pH 1.0–12.0) at 37 °C for 1 h, and the residual enzyme activity was detected under standard conditions (pH 4.5, 65 °C and 10 min). The buffers used were 100 mM glycine–HCl (pH 1.0–3.0), McIlvaine buffer (pH 2.5–8.0), 100 mM Tris–HCl (pH 8.0–9.0) and 100 mM glycine–NaOH (pH 9.0–12.0).

Thermal stability was determined by measuring the residual enzyme activity after incubation at 55–90 °C for 30 min and the half-life of enzyme inactivation (*t*_1/2_) at 65–80 °C and optimal pH. The residual enzyme activities were measured under standard conditions.

To evaluate the effect of metal ions and chemical reagents on the glucanase activity of purified recombinant *Tl*Glu16A, different salts (KCl, NaCl, CaCl_2_, CoCl_2_, MgSO_4_, NiSO_4_, CrCl_3_, CuSO_4_, FeCl_3_, MnSO_4_, AgNO_3_, Pb(CH_3_COO)_2_, ZnSO_4_ and HgCl_2_) (5 and 10 mM) and chemical reagents (EDTA, SDS and β-mercaptoethanol) (5 and 10 mM) were added to the assay system individually. The relative activities were determined against the activity of the control sample without any addition [39].

Substrate specificity of *Tl*Glu16A was assayed at 65 °C for 10 min in McIlvaine buffer (pH 4.5) containing 1.0 % (w/v) barley β-glucan, lichenan, laminarin, birch wood, xylan, Avicel, CMC-Na, *p*NPG or *p*NPC. The kinetic parameters *K*_m_ and *V*_max_ were determined in McIlvaine buffer (pH 4.5) containing 0.25–5.0 mg/mL of barley β-glucan and lichenan for 5 min. The kinetic values were determined by fitting the Lineweaver–Burk plot [56].

The hydrolysis products of barley β-glucan and lichenan by *Tl*Glu16A were determined using the TLC. Each enzyme (50 U) was added into 1 mL of 1 % (w/v) of each substrate in 50 mM Na_2_HPO_4_–citric acid (pH 4.5) and incubated at 55 °C for 1 h. Aliquots were withdrawn at different time intervals and boiled for 5 min to terminate the reactions. The hydrolysis products were spotted on a silica gel plate (model 60F254; Merck, Darmstadt, Germany). The plate was developed with two runs in a solvent system of butanol:acetic acid:water (2:1:1, v/v/v). After spraying with methanol:sulfuric acid (95:5, v/v) solvent, the sugars on the plate were visualized by heating for a few minutes at 130 °C. A cellooligosaccharide mixture consisting of glucose (G1), cellobiose (G2), cellotriose (G3), cellotetraose (G4) and cellopentaose (G5) was used as the standard.

### ETSS calculation and site-directed mutagenesis

The total interaction energy (*E*_*ij*_) between charged amino acids of *Tl*Glu16A was calculated using the ETSS [57, 58]. Any mutation that changes the E_ij_ value from positive to negative values may improve the structural stability of *Tl*Glu16A. According to the combined strategy of potential mutation site selection [33], multiple-sequence alignment and structure modeling (PyMOL; Delano Scientific, Portland, OR), charged residues were modified with oppositely charged ones and otherwise to neutral Ala.

Site-directed mutagenesis was conducted by overlap extension PCR [33] with the gene fragment of *Tlglu16A* as the DNA template and the primers listed in additional files 1, 2, 3 and 4. The PCR products were ligated to the vector pEASY-T3 for sequencing. The mutants harboring the correct mutations were digested by restriction endonucleases, expressed in *P. pastoris* and characterized as described above.

### Thermal properties of the wild-type *Tl*Glu16A and its mutants

Thermostability was determined by measuring the half-life of enzyme inactivation (*t*_1/2_) at 65–80 °C and each optimum pH. All purified enzymes were diluted to 100 mg/mL in McIlvaine buffer and incubated for different durations (TlGlu16A (pH 4.5), H58D (pH 4.5), E134R (pH 4.0), D235G (pH 4.0) and D296K (pH 4.0)). The residual enzyme activities were measured under optimal conditions of each enzyme (TlGlu16A (pH 4.5 and 65 °C), H58D (pH 4.5 and 70 °C), E134R (pH 4.0 and 65 °C), D235G (pH 4.0 and 70 °C) and D296K (pH 4.0 and 70 °C)). To determine the thermal tolerance (temperature at which 50 % of activity is lost [*T*_50_]), wild-type *Tl*Glu16A and mutants were diluted to 50 μg/mL in McIlvaine buffer (TlGlu16A (pH 4.5), H58D (pH 4.5), E134R (pH 4.0), D235G (pH 4.0) and D296K (pH 4.0)) and then heated for 30 min at temperatures ranging from 55 to 90 °C at intervals of 5 °C. After heating, the enzymes were immediately placed on ice for 10 min, followed by the residual glucanase activity assay as described above. All reactions were performed in triplicate.

### DSC analysis

The melting temperature, *T*_m_, was determined by a Nano-DSC (TA Instruments, New Castle, DE). Each protein sample, 350 μg, was dissolved in 1 mL of 10 mM PBS (pH 6.8). The same amount of buffer was used as the blank control. The degassed protein samples and control were loaded into the calorimeter cells and treated with a heating rate of 1 °C/min over the temperature range of 25–100 °C. The transition state was scanned at the rate of 1 °C/min. Each experiment was repeated at least three times.

### Enzymatic hydrolysis of corn stover

Corn stover was treated with 15 % (w/w) ammonia solution for 24 h as previously reported [9]. Wild-type *Tl*Glu16A and mutant H58D (each at 0.06 μmol) were each incubated in 100 mM sodium citrate (pH 4.8) containing 5 % (*w*/*v*) corn stover at 50 °C and 165 rpm for 60 h. The reducing sugars released were determined every 12 h using the DNS method. The experiment was performed in triplicate.

### Nucleotide sequence accession number

The nucleotide sequence of the endoβ-1,3-1,4-glucanase gene (*Tlglu16A*) from *T. leycettanus* JCM12802 was deposited in the GenBank database under accession no. KU194473.

## Additional files

10.1186/s13068-016-0544-8 Multiple sequence alignment of deduced *Tl*Glu16A and five other fungal counterparts of GH16.
10.1186/s13068-016-0544-8 SDS-PAGE analysis of purified recombinant *Tl*Glu16A and its mutants. Lanes: M, the standard protein molecular weight markers; G1, the crude enzyme of wild-type *Tl*Glu16A; G2, 2, 4, 6 and 8, the purified *Tl*Glu16A and mutants H58D, E134R, D235G and D296K, respectively; G3, 1, 3, 5 and 7, the deglycosylated *Tl*Glu16A and mutants H58D, E134R, D235G and D296K, respectively.
10.1186/s13068-016-0544-8 Total interaction energies of *Tl*Glu16A determined by ETSS.
10.1186/s13068-016-0544-8 Oligonucleotide primers used in this study.

## Abbreviations

CMC-Na: carboxymethyl cellulose-sodium
TAIL-PCR: thermal asymmetric interlaced-PCR
SDS-PAGE: sodium dodecyl sulfate-polyacrylamide gel electrophoresis
DNS: 3,5-dinitrosalicylic acid
RT-PCR: reverse-transcription PCR
ORF: open reading frame
LC/ESI-TOF-MS: liquid chromatography/electrospray ionization tandem time-of-flight mass spectrometry
MALDI-TOF: matrix-assisted laser desorption/ionization time-of-flight
ETSS: enzyme thermal stability system
MD: minimal dextrose medium
BMGY: buffered glycerol-complex medium
BMMY: buffered methanol-complex medium
TLC: thin layer chromatography
Endo H: endo-β-*N*-acetylglucosaminidase H
*p*NPG: 4-nitrophenyl α-d-galactopyranoside
*p*NPC: 4-nitrophenyl β-d-cellobioside
DSC: differential scanning calorimetry
