# Supplementary material for: Improvement of the thermostability and catalytic efficiency of a highly active β-glucanase from Talaromyces leycettanus JCM12802 by optimizing residual charge–charge interactions
Source: Biotechnol Biofuels. 2016 Jun 13;9:124. doi: 10.1186/s13068-016-0544-8 (PMC4906821; doi:10.1186/s13068-016-0544-8)
Supplement: Supplementary file 1 — 10.1186/s13068-016-0544-8 Multiple sequence alignment of deduced TlGlu16A and five other fungal counterparts of GH16. [file 13068_2016_544_MOESM1_ESM.doc]

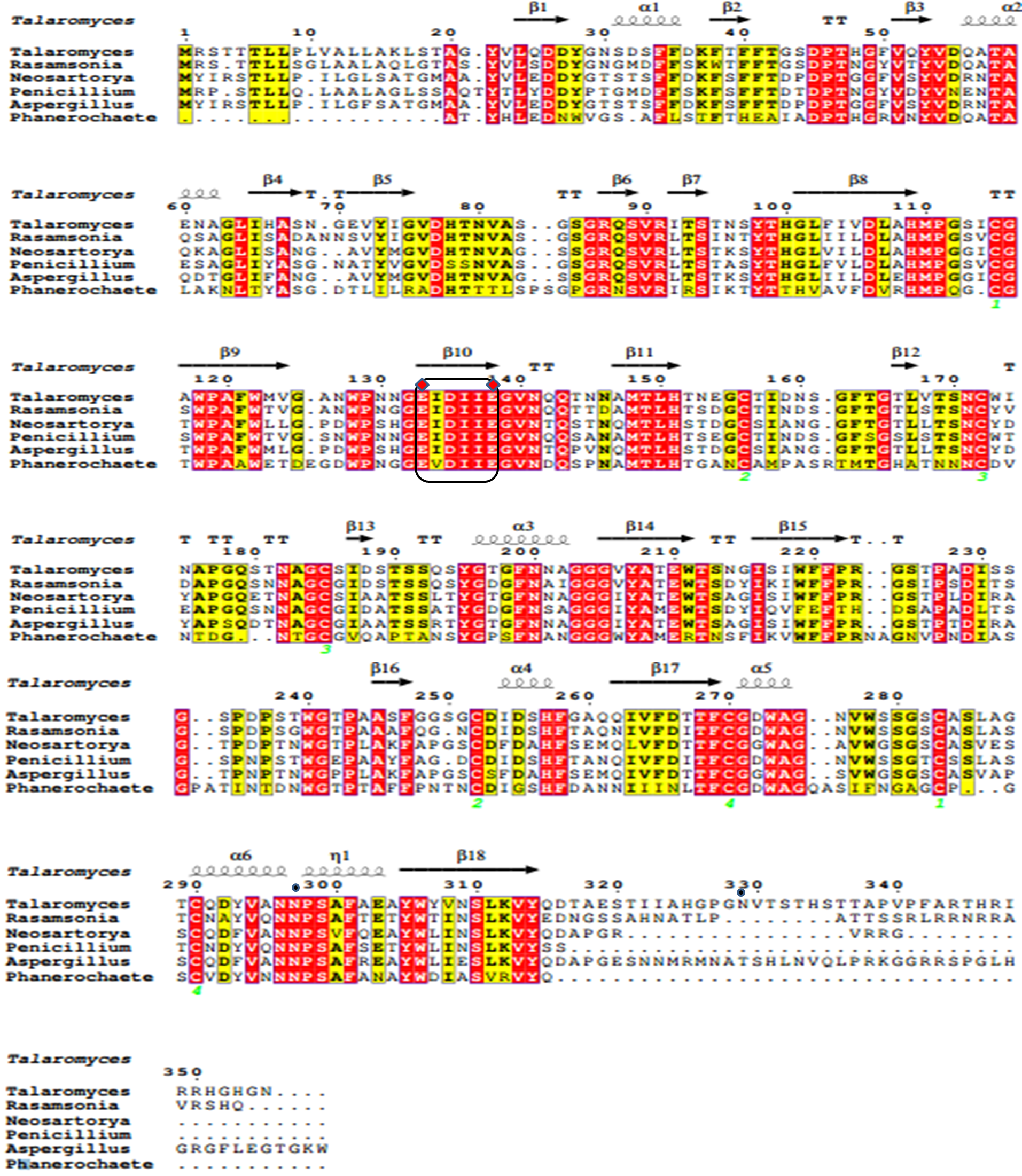


**Additional file 1: Multiple sequence alignment of deduced *Tl*Glu16A and other fungal counterparts of GH16.**
