# Supplementary material for: Improvement of the thermostability and catalytic efficiency of a highly active β-glucanase from Talaromyces leycettanus JCM12802 by optimizing residual charge–charge interactions
Source: Biotechnol Biofuels. 2016 Jun 13;9:124. doi: 10.1186/s13068-016-0544-8 (PMC4906821; doi:10.1186/s13068-016-0544-8)
Supplement: Supplementary file 2 — 10.1186/s13068-016-0544-8 SDS-PAGE analysis of purified recombinant TlGlu16A and its mutants. Lanes: M, the standard protein molecular weight markers; G1, the crude enzyme of wild-type TlGlu16A; G2, 2, 4, 6 and 8, the purified TlGlu16A and mutants H58D, E134R, D235G and D296K, respectively; G3, 1, 3, 5 and 7, the deglycosylated TlGlu16A and mutants H58D, E134R, D235G and D296K, respectively. [file 13068_2016_544_MOESM2_ESM.doc]

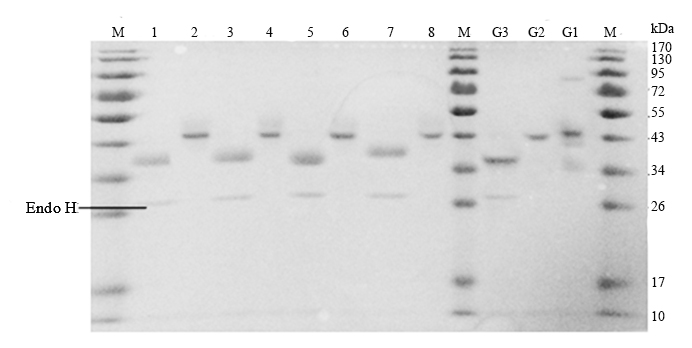


**Additional file 2: SDS-PAGE analysis of purified recombinant *Tl*Glu16A and its mutants.** Lanes: M, the standard protein molecular weight markers; G1, the crude enzyme of wild type *Tl*Glu16A; G2, 2, 4, 6 and 8, the purified *Tl*Glu16A and mutants H58D, E134R, D235G and D296K, respectively; G3, 1, 3, 5 and 7, the deglycosylated *Tl*Glu16A and mutants H58D, E134R, D235G and D296K, respectively.
