# Supplementary figures and images for: Improvement of the thermostability and catalytic efficiency of a highly active β-glucanase from Talaromyces leycettanus JCM12802 by optimizing residual charge–charge interactions
Source: Biotechnol Biofuels. 2016 Jun 13;9:124. doi: 10.1186/s13068-016-0544-8 (PMC4906821; doi:10.1186/s13068-016-0544-8)

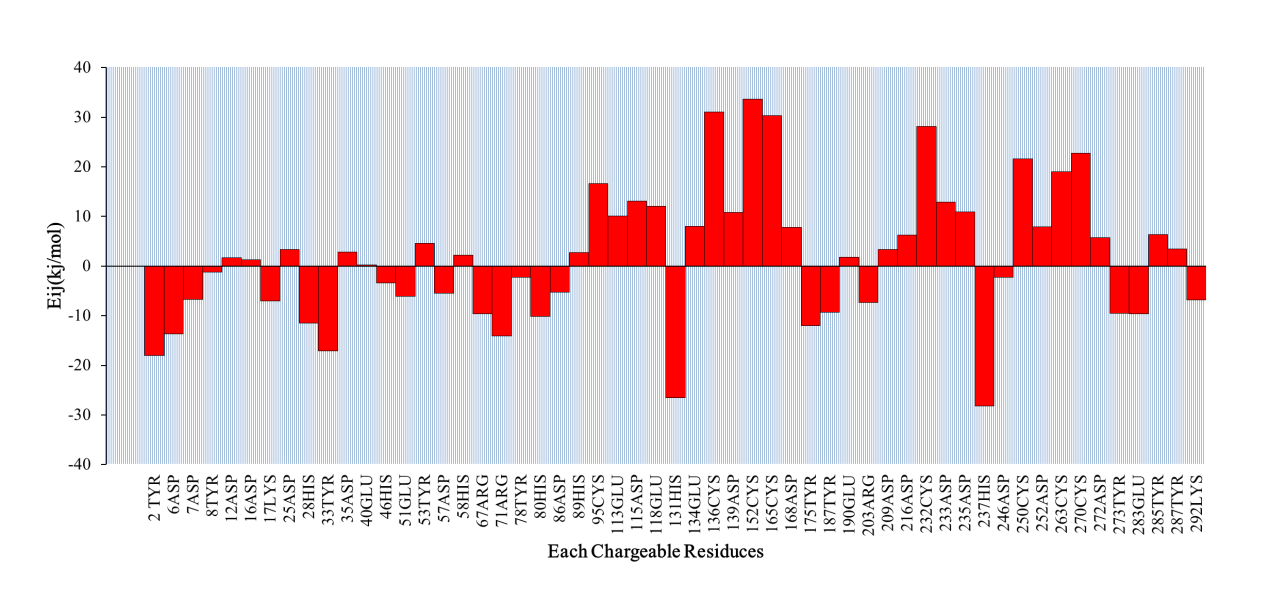


**Additional file 3: Total interaction energies of *Tl*Glu16A determined by ETSS**

Supplement: Supplementary file 3 — 10.1186/s13068-016-0544-8 Total interaction energies of TlGlu16A determined by ETSS. [file 13068_2016_544_MOESM3_ESM.doc]
