# Supplementary material for: Improvement of the thermostability and catalytic efficiency of a highly active β-glucanase from Talaromyces leycettanus JCM12802 by optimizing residual charge–charge interactions
Source: Biotechnol Biofuels. 2016 Jun 13;9:124. doi: 10.1186/s13068-016-0544-8 (PMC4906821; doi:10.1186/s13068-016-0544-8)
Supplement: Supplementary file 4 — 10.1186/s13068-016-0544-8 Oligonucleotide primers used in this study. [file 13068_2016_544_MOESM4_ESM.doc]

**Additional file 4: Oligonucleotide primers used in this work**

| **Primers** | **Sequences**  **(5′→3′) ^a^** | **Size (bp)** | ***T*_m_**  **(°C) ^b^** |
| --- | --- | --- | --- |
| GH16F | TGCGGTAYNTGGCCNGC | 17 | 45.0–50.0 |
| GH16R | CCGGCCCANTBNCCRCARAA | 20 | 45.0–50.0 |
| dsp1 | CCTGACCTGGCAACAGCTGGATGGT | 25 | 60.5 |
| dsp2 | TGGCTTCACGGGCACTCTCGTCAC | 24 | 60.5 |
| dsp3 | ATCGACCTGGGGTACTCCCGCG | 22 | 60.6 |
| usp1 | AGTACCCCAGGTCGATGGGTCGG | 23 | 60.6 |
| usp2 | CCGTGCCGTAGGACTGTGAGGAG | 23 | 60.6 |
| usp3 | CCGTTGTTCGGCCAGTTGGCACC | 23 | 60.6 |
| Tlglu16A-F | ATGCGGTCCACAACCACACTCCTT | 24 | 57.1 |
| Tlglu16A-R | CTAATTGCCATGGCCATGTCTGCG | 24 | 57.1 |
| D16A-F | GGATTCCTTCTTTGCCAAGTTCACTTTC | 28 | 56.0 |
| D16A-R | GAAAGTGAACTTGGCAAAGAAGGAATCC | 28 | 56.0 |
| E40R-F | GGCCACAGCACGGAATGCAGG | 20 | 58.7 |
| E40R-R | CCTGCATTCCGTGCTGTGGCC | 21 | 58.7 |
| H58D-F | GGCGTCGATGATACCAATGTCGC | 23 | 57.0 |
| H58D-R | GCGACATTGGTATCATCGACGCC | 23 | 57.0 |
| E134R-F | CACACTAACCGGGGATGCACC | 21 | 56.7 |
| E134R-R | GGTGCATCCCCGGTTAGTGTG | 21 | 56.7 |
| D139A-F | GCACCATCGCCAACTCTGGC | 20 | 56.6 |
| D139A-R | GCCAGAGTTGGCGATGGTGC | 20 | 56.6 |
| E190R-F | GCGTCTACGCCACCCGGT | 18 | 56.2 |
| E190R-R | GTTGCTGGTCCACCGGGTG | 19 | 56.4 |
| D216A-F | GCCCCGCCCCATCGAC | 16 | 55.7 |
| D216A-R | GTCGATGGGGCGGGGC | 16 | 55.7 |
| D233H-F | CAGGCTGCCACATCGACTCG | 20 | 56.6 |
| D233H-R | CGAGTCGATGTGGCAGCCTG | 20 | 56.6 |
| D235G-F | GCGACATCGGCTCGCACTTTG | 21 | 56.7 |
| D235G-R | CAAAGTGCGAGCCGATGTCGC | 21 | 56.7 |
| D272K-F | CACATGCCAGAAGTACGTCGCC | 22 | 56.9 |
| D272K-R | GGCGACGTACTTCTGGCATGTG | 22 | 56.9 |
| D296K-F | GGTCTACCAGAAGACCGCGG | 20 | 56.6 |
| D296K-R | CCGCGGTCTTCTGGTAGACC | 20 | 56.6 |
| Tlglu16AF-s | GACGAATTCGGTTATGTGCTGCAGGATGATTATGGAAAC | 39 | 58.0 |
| Tlglu16AR-s | GACGCGGCCGCCTAATTGCCATGGCCATGTCTGCG | 35 | 57.0 |

^a^Y = C/T , R = A/G, B = T/G/C, N = A/T/G/C; restriction sites are underlined.

^b^*T*_m_: Annealing temperature.
